# Vanadium Pentoxide Nanobelt-Reduced Graphene Oxide Nanosheet Composites as High-Performance Pseudocapacitive Electrodes: *ac* Impedance Spectroscopy Data Modeling and Theoretical Calculations

**DOI:** 10.3390/ma9080615

**Published:** 2016-07-25

**Authors:** Sanju Gupta, Bryce Aberg, Sara B. Carrizosa, Nicholas Dimakis

**Affiliations:** 1 Department of Physics and Astronomy and Advanced Materials Institute, Western Kentucky University, Bowling Green, KY 42101, USA; 2 Department of Electrical Engineering, Western Kentucky University, Bowling Green, KY 42101, USA; bryce.aberg543@topper.wku.edu; 3 Department of Chemistry, Western Kentucky University, Bowling Green, KY 42101, USA; sara.boterocarrizosa090@topper.wku.edu; 4 Department of Physics, The University of Texas-Rio Grande Valley, Edinburg, TX 78539, USA; nicholas.dimakis@utrgv.edu

**Keywords:** graphene, vanadium pentoxide, pseudocapacitors, impedance spectroscopy analysis, modeling, DFT calculations

## Abstract

Graphene nanosheets and graphene nanoribbons, G combined with vanadium pentoxide (VO) nanobelts (VNBs) and VNBs forming GVNB composites with varying compositions were synthesized via a one-step low temperature facile hydrothermal decomposition method as high-performance electrochemical pseudocapacitive electrodes. VNBs from vanadium pentoxides (VO) are formed in the presence of graphene oxide (GO), a mild oxidant, which transforms into reduced GO (rGO_HT_), assisting in enhancing the electronic conductivity coupled with the mechanical robustness of VNBs. From electron microscopy, surface sensitive spectroscopy and other complementary structural characterization, hydrothermally-produced rGO nanosheets/nanoribbons are decorated with and inserted within the VNBs’ layered crystal structure, which further confirmed the enhanced electronic conductivity of VNBs. Following the electrochemical properties of GVNBs being investigated, the specific capacitance *C*_sp_ is determined from cyclic voltammetry (CV) with a varying scan rate and galvanostatic charging-discharging (*V–t*) profiles with varying current density. The rGO-rich composite V_1_G_3_ (i.e., VO/GO = 1:3) showed superior specific capacitance followed by VO-rich composite V_3_G_1_ (VO/GO = 3:1), as compared to V_1_G_1_ (VO/GO = 1:1) composite, besides the constituents, i.e., rGO, rGO_HT_ and VNBs. Composites V_1_G_3_ and V_3_G_1_ also showed excellent cyclic stability and a capacitance retention of >80% after 500 cycles at the highest specific current density. Furthermore, by performing extensive simulations and modeling of electrochemical impedance spectroscopy data, we determined various circuit parameters, including charge transfer and solution resistance, double layer and low frequency capacitance, Warburg impedance and the constant phase element. The detailed analyses provided greater insights into physical-chemical processes occurring at the electrode-electrolyte interface and highlighted the comparative performance of thin heterogeneous composite electrodes. We attribute the superior performance to the open graphene topological network being beneficial to available ion diffusion sites and the faster transport kinetics having a larger accessible geometric surface area and synergistic integration with optimal nanostructured VO loading. Computational simulations via periodic density functional theory (DFT) with and without V_2_O_5_ adatoms on graphene sheets are also performed. These calculations determine the total and partial electronic density of state (DOS) in the vicinity of the Fermi level (i.e., higher electroactive sites), in turn complementing the experimental results toward surface/interfacial charge transfer on heterogeneous electrodes.

## 1. Introduction

Intense research activity on sustainable renewable energy is stimulated by the increasingly global demand of electric energy. Electrochemical energy storage systems (EES), namely electrochemical super- (SCs) and pseudo-capacitors (PCs) and rechargeable secondary batteries, represent the most efficient and environmentally-benign technologies. The need for next-generation stable, high-performance, cost-effective electrode materials (higher energy and power density with longer cycle life) and novel architectures is the driving force for addressing the scarcity of energy availability worldwide while mitigating environmental pollution. While energy is stored in supercapacitors by forming an electrochemical double layer by the adsorption of electrolyte ions on the electrode surface, the energy storage in pseudocapacitors is through actual electron transportation, unlike rechargeable secondary batteries (Faradaic redox reactions) via the conversion between chemical energy into electricity. However, it is noteworthy that in practical electrochemical electrodes, the two energy storage mechanisms often work concomitantly. Nanotechnology has accelerated the innovation of novel electrode formulations to replace conventional carbon-based nanomaterials as supercapacitive cathodes and transition metal oxides as pseudocapacitive cathodes by themselves, which calls for hybrids or composites. Hybrid composites specifically comprising metal-graphene interfaces are generally developed for several electronic, electrochemical, electroanalytical and biological applications. Graphene, which is a seamless sheet of sp^2^-bonded carbon (sp^2^ C) atoms, has excellent physical-chemical properties, traditionally created for nanoelectronics and, more recently, is an emerging candidate for electrochemical energy storage systems due to the excellent specific surface area (~theoretical ca. 2630 m^2^/g), higher electrical conductivity and mechanical strength. Likewise, graphene derivatives, including graphene oxide (GO), with various surface functional groups, and its reduced form (rGO), prepared using different reduction methods, are equally promising for various technologies. In general, epoxide (–O–) and carboxyl (–COOH) groups located on the basal plane of the GO nanosheet are major components; carbonyl (–C=O) and hydroxyl (–OH) groups distributed at the edges of GO sheets are minor components. Preferential reduction of basal plane moieties leaving residual and random edge functional sites are produced using chemical (rGO), electrochemical (ErGO) and thermal (rGO_HT_) reduction methods [1]. Transition metal oxides (TMeO_*x*_)/hydroxides [2,3,4], such as RuO_*x*_, Mn_*x*_O_*y*_, Co_*x*_O_*y*_, V_*x*_O_*y*_, FeO_*x*_, NiO, Ni(OH)_2_ and Co(OH)_2_, exhibited attractive performance for pseudocapacitor applications. In spite of extensive exploitation of diverse nanocarbons and transition metal oxides as electrochemical electrodes, they are limited due to insufficient electrochemical activity, cycle lifetime and high cost. The continued interest in the strategic combination of TMeO_*x*_ and low-dimensional carbons is relevant, since it generates synergistic and complementary effects, yielding versatile properties to fit new functions for applied electrochemistry (hybrid supercapacitive cathodes, i.e., with a pseudocapacitive contribution, battery anodes, electroanalytical and biosensing) [5,6,7,8]. Alternatively, multifunctional hybrid supercapacitive electrode assembly with strongly coupled or chemical bridged inorganic/nanocarbons interfaces promotes effective surface charge transfer sites and faster electron/ion transport during charging-discharging cyclability [9,10,11,12]. Furthermore, they are actively pursued due to their complementary (i.e., coexistent specific energy and power densities, long cycle life, wider potential window and thermal operating range, low maintenance cost) features as opposed to rechargeable secondary lithium-ion batteries and fuel cells, for instance [13,14,15,16,17,18,19]. 

Among known pseudocapacitive materials offering higher energy density, vanadium pentoxide (V_2_O_5_; VO) has been widely investigated as a potential candidate material, because of being a low-cost, abundant resource, having a layered structure, high energy density and wide potential window arising from its multivalent oxidation states (V^3+^/V^4+^/V^5+^) [16,20,21,22,23,24]. This work presents the development of reduced graphene oxide (rGO) as a two-dimensional nanomaterial in combination with nanostructured VO forming nanocomposite electrodes as high-performance pseudocapacitors. Since bulk VO has limited performance in a device owing to poor electronic conductivity, it requires nanostructuring, such as nanowires, nanotubes and nanobelts, where the latter are demonstrably promising high-performance electrochemical electrodes as supercapacitors and Li-ion battery cathodes delivering energy to load on-demand at the system level [25,26,27]. In this regard, we prepared nanocomposites comprising rGO, G nanosheets and VO nanobelts (VNBs), i.e., GVNBs, using a facile low temperature single-step hydrothermal technique devoid of harsh chemicals and reducing agents unlike other time-consuming complex synthesis approaches. The nanostructured VO involves redox reactions (pseudocapacitance), which provides higher power and energy density, and highly conducting rGO, having a large surface area, which implies supercapacitive behavior. We argue that these approaches are anticipated to facilitate higher ion adsorption and surface charge transfer (interfacial activity) due to chemical bridging between graphene support and pseudocapacitive V_*x*_O_*y*_, augmenting electrochemical (re)activity and, thus, energy storage. Although several studies have shown progress [21,28,29,30,31], a comprehensive demonstration meeting relevant criteria for practical devices has yet to be presented. Through extensive evaluation for a set of materials using different approaches presented in this work, their full potential as electrochemical electrodes combined with fundamental insights is established. The findings are discussed in terms of: (1) nanoscale control of reactivity, such as the presence of low-coordinated sites in multi-materials; and (2) theoretical calculations, highlighting the interfacial charge transfer through chemical bridging that allows available electron density of states, albeit small in the vicinity of the Fermi level. The knowledge gained in this work can tap into Generation II or next-generation scalable high-performance electrochemical energy storage and sensing platforms.

## 2. Methodology, Materials, Methods and Characterization

### 2.1. Hydrothermal Synthesis and Electrode Preparation

**Figure 1:**
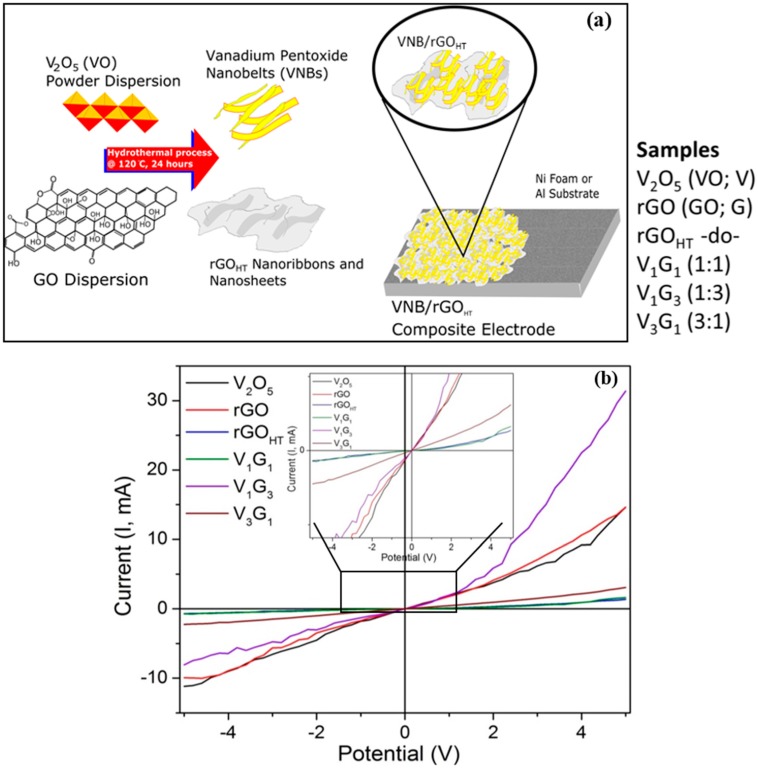
(**a**) Schematic of the composite electrodes comprised of vanadium pentoxide (VO) nanobelts (VNBs) and rGO nanosheets/nanoribbons in different composition ratios using hydrothermal synthesis technique; (**b**) room temperature *I*–*V* electrical properties showing quasi-linear (or non-ohmic) behavior.

Graphene oxide (GO) was prepared by the modified Hummer’s method followed by chemical reduction using hydrazine monohydrate, producing reduced GO (rGO) [32]. The hydrothermal technique is gaining popularity and gathering interest from scientists and technologists of different disciplines [33,34]. Briefly, micro-particles of V_2_O_5_, chemically-reduced graphene oxide (rGO), hydrothermally-processed rGO (rGO_HT_) and composites of rGO_HT_ (from GO) mixed with VO micro-particles at VO/GO ratios of 1:1, 1:3 and 3:1 were synthesized. The rGO_HT_ and composites (VO/GO) in the form of powders were prepared using a one-step hydrothermal process, where dispersions of mixed materials (1 mg/mL GO and 0.1 mg/mL VO) with DI water were placed in a Teflon-lined autoclave and placed in a box furnace at constant temperature for a certain deposition time (see Table 1 for details on the deposition parameters for each electrode material). The dispersed solutions prepared in a furnace were poured into a micro-filter (<20 μm) and allowed to dry in air for 48 h, such that only powder is retained. In total, six different materials are prepared and transferred to two different substrates as electrodes. Figure 1a shows the schematic of the deposition scheme. During the synthesis, VO microparticles were converted into uniformly-distributed nanobelts with simultaneous reduction of GO into rGO. Under hydrothermal conditions, GO may act as a mild oxidizing agent to synthesize VO nanobelts in DI water. The substrates were 1.5 × 5 cm^2^ of aluminum foil and nickel foam that were cleaned in acetone and dried with N_2_. To prepared electrodes, a slurry comprised of 80 wt % active electrode material, 10 wt % PVDF (polyvinylidene fluoride) as a binder and 10 wt % carbon black was formed by mixing these components with NMP (*N*-methyl-2-pyrrolidone) in a mortar and pestle. The slurry was applied to cleaned substrates using a blade knife, and they were left to dry at room temperature for at least 24 h. After the electrodes had dried, they were placed into a quartz tube furnace in Ar atmosphere for annealing at 120 °C for 4 h.

### 2.2. Sample Characterization

All of the samples were characterized in terms of surface morphology and microstructure, crystal structure and electrochemical properties. Scanning electron microscopy (SEM) images were taken with an instrument (Model JEOL 6010Plus, Peabody, MA, USA) operating at primary electron acceleration voltage (*V*_acc_) of 10 kV and a constant current of 45 μA in secondary electron imaging (SEI) mode collected with an in-lens detector equipped with an X-ray ISIS EDS system providing surface morphology. Samples for TEM (transmission electron microscopy) and SAED (selected area electron diffraction) were prepared by placing one to two drops of individual component and of hybrids on commercial lacey carbon-coated Cu mesh grids (Ted Pella Inc., Redding, CA, USA) and allowed to air dry. They were taken using a JEOL instrument (JEOL Model 1400 Plus, Peabody, MA, USA) operating in cryo-EM and SAED modes at 200 kV or 100 kV and 1 nA with a Be specimen holder, a Gresham SiLi detector with a Moxtek AP3.3 window. For SAED patterns, we used a 0.20-μm aperture, producing a small spot size, and spread the beam to increase the electron coherence length at the samples. TEM and SAED measurements provided intrinsic microstructure and nanoscale morphology and determined interplanar spacing. X-ray diffraction (XRD) patterns were obtained with the Siemens Model D5000 instrument (now Thermo Scientific, Waltham, MA, USA) in Bragg–Brentano θ-2θ geometry ranging 2θ from 8° to 60° using a Cu K_α_ X-ray source (λ = 1.5405 Å) operating at a voltage of 45 kV and a current of 40 mA. Samples were run at a scan rate of 0.04°/s or to improve the signal-to-noise ratio; a scan rate of 0.02°/s was used. Raman spectra were measured to determine the lattice vibration at various regions of interest on the samples. The spectra were recorded using a micro-Raman spectrometer (Model InVia Renishaw *plc*, Gloucestershire, UK) equipped with a laser providing an excitation wavelength of 633 nm (*E*_L_ = 1.92 eV) and an ~4–6-mW power incident on the sample, with the edge filter cutting at ~100 cm^−1^. The scattered light from the sample is collected in backscattering geometry transmitted by a beam splitter and detected by a CCD camera. The reflected light is filtered using an edge filter to remove the laser excitation and sent to a spectrometer. An objective lens of 50× was used providing a spot size of ~1–2 μm, and extreme care was taken to avoid sample damage or laser-induced thermal degradation. Either 5% or 10% light intensity was used to accurately obtain spectra while avoiding thermal degradation. Spectra were acquired at time intervals of 30–60 s, although increased to a few minutes to maximize signal throughput. The Raman shift ranged from 150 to 3200 cm^−1^ for hybrid composites with a spectral resolution of 1 cm^−1^. Allowing more details and understanding the interaction between rGO_HT_ and VNB, we measured room temperature electrical (*I*–*V*) properties’ in-plane configurations. We made electrical contacts with colloidal silver paste and attached a Cu wire for connection with the Keithley 2400 source meter (Keithley, Cleveland, OH, USA). We measured in-plane two-point contact resistance and determined room temperature electrical conductivity (σ_dc_) for all of the samples studied. The hybrids apparently displayed semiconducting (i.e., non-ohmic or nonlinear) behavior, as anticipated. Quantitatively, the electrical conductivity measured for these hybrids ranged from 0.01 to 2.1 s/cm (see Figure 1b).

Electrochemical tests were performed on those samples prepared on nickel foam substrates using an electrochemical bi-potentiostat workstation (Model 920D, CH Inc., Austin, TX, USA) including cyclic voltammetry (CV), galvanostatic charging-discharging *V–t* profiles, cyclability and *ac* electrochemical impedance spectroscopy (EIS). CV was conducted in the potential window of 0.0–0.8 V at scan rates between 2.5 and 200 mV/s in 0.5 M sodium sulfate (Na_2_SO_4_) electrolyte using a Ag/AgCl reference electrode (RE), Pt wire as the counter electrode (CE) and the working electrode (WE) as prepared samples. Electrochemical impedance spectroscopy (*ac* EIS) was performed at open circuit voltage and +0.2 V with superimposed 5 mV ac signal within a frequency range from 0.01 Hz to 98 kHz. Galvanostatic charging-discharging tests were conducted using cathode and anode currents ranging between 2.5 and 150 mA (or 0.5 A·g^−1^ and 30 A·g^−1^). Each test allowed the cell to charge for 10 s or until its voltage reached ~1 V, at which point, the cell began discharging, and this cycle was repeated >500 times.

Density functional theory is employed to determine the density of state s and charge transfer between graphene and VO. Briefly, a monolayer of graphene is modeled as a two-dimensional hexagonal lattice with a calculated lattice constant of 2.45 Å, which is slightly lower than the corresponding experimentally-measured value for graphite reported at 2.46 Å [35]. The adatom-substrate systems were modeled with adatoms adsorbed on (4×4) graphene supercells. The CRYSTAL09 [36] program that employs Gaussian type basis set functions centered at the atoms was used for periodic DFT graphene calculations with and without adatoms. Restricted (constrained relaxation) and unrestricted (relaxed) DFT with the hybrid PBE0 (Perdew–Burke–Ernzerhof) non-empirical/parameter-free function [37,38] was employed to focus on the interface between vanadium oxides clusters and graphene nanosheets (GNS). All atoms are treated by all-electron basis sets from Peintinger et al. [39], which are optimized for crystalline calculations (pob-TZVP, triple zeta valence potential with polarization basis set). These basis sets are developed from the original triple-ζ for valence plus single polarization function basis set by Ahlrichs [40,41] (def2-TZVP basis set) through the reduction in the number of Gaussians used (e.g., for carbon atoms, the original def2-TZVP basis set ([5s3p2d1f] is contracted to [4s3p1d]). Brillouin zone integrations were performed on a 24 × 24 Monkhorst–Pack grid [42], which is also used for Fermi energy (*E*_F_) and the density matrix calculations (Gilat grid) [43,44]. The Fermi surface was smeared with a Gaussian of 0.005 Hartrees for convergence purposes. Moreover, the self-consistent field (SCF) energy convergence was achieved by using Anderson quadratic mixing [45], coupled with additional mixing of the occupied orbitals with the virtual orbitals. The SCF energy threshold value for our calculations was set at 10^−9^ Hartrees for clean graphene substrates and the adatom-graphene systems to 10^−7^ Hartrees, suggesting covalent and electrostatic bonding.

## 3. Results and Discussion

### 3.1. Microscopic Structural Characterization

**Figure 2:**
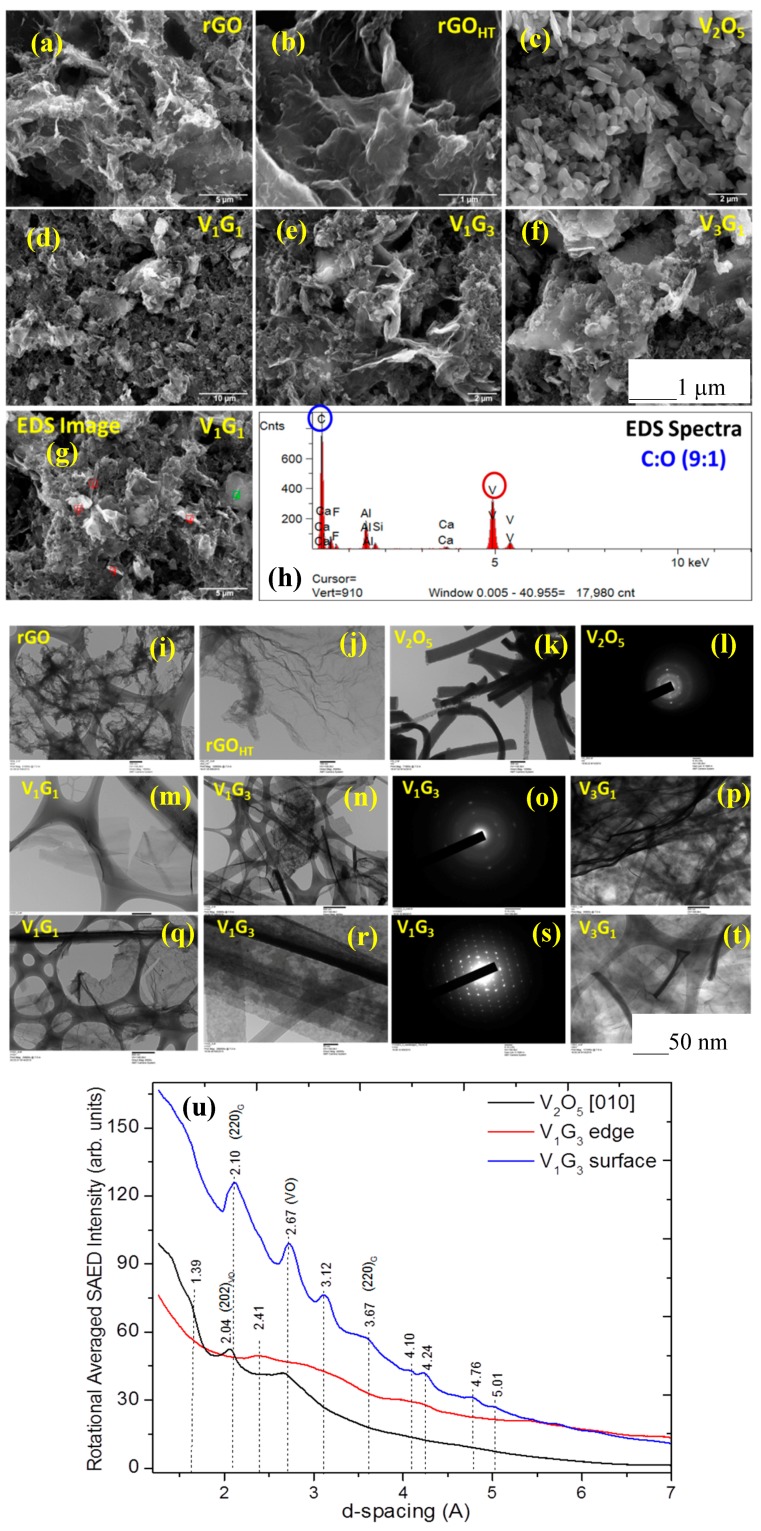
(**a**–**g**) SEM micrographs; (**h**) EDS spectra; TEM micrographs of (**i**) rGO; (**j**) rGO_HT_; (**k**) V_2_O_5_; (**m**,**q**) V_1_G_1_; (**n**,**r**) V_1_G_3_; (**p**,**t**) V_3_G_1_; (**l**,**o**,**s**) SAED and (**u**) the real space (d_hkil_) intensity pattern revealing the crystal structure of ‘hybrids’ for the representative samples studied. The surface morphology and semicrystalline nature of VNBs with rGO_HT_ nanosheets is apparent (scale bars are shown at the bottom of the images).

Figure 2a–g presents representative SEM images of rGO, rGO_HT_, crystalline VNB and their composites along with the EDS image and elemental spectra (Figure 2h) for the V_1_G_1_ composite. During the hydrothermal synthesis, VO microparticles were converted into uniformly-distributed nanobelts with simultaneous reduction of GO into rGO_HT_. Moreover, under hydrothermal conditions of lower temperature (<200 °C) and pressure (greater than a few atmospheres) in an autoclave, GO with oxygenated surface functionalities acts as a mild oxidizing agent to promote the synthesis of nanostructured VO (e.g., VNBs) in DI water. They reveal a relatively uniform surface morphology by themselves and as hybrid composites, wherein the interconnected network and crumpled silk-liked structured graphene nanosheets (GNS) as larger nanoribbons are uniformly blended with VNBs with a lateral size range of 50–100 nm, which could prevent the commonly-observed restacking of GNS. To investigate the structural information of the components and hybrids at the nanoscale, TEM combined with SAED results are presented in Figure 2i–t. HRTEM images apparently show polycrystalline tubular or cylindrical VNBs with size ranging from 20 to 50 nm and graphene nanoribbons of 510 nm interfacing with the edge of VNBs. Structural order is evident from the SAED and intensity patterns, which are associated with the structural signatures of crystalline VNBs and decorated GNS with VNB. SAED intensity versus interplanar spacing (*d*_hkl_) (Figure 2u) reveals the cubic lattice crystal structure for VNB (marked), and some of the additional peaks in all of the composites are attributed to graphene (marked), at spacings of 0.267 ± 0.01 nm (002) and 0.204 ± 0.01 nm (202) peaks [46,47]. The elements C, V and O are identified, and the loading of V_*x*_O_*y*_ is determined using EDS, shown in Figure 2h. The EDS showed that the C to O ratios are uniform across most of the samples, as expected for a single phase of 9:1, indicative of the reduction of GO during hydrothermal reduction, producing rGO_HT_, and the optimal V to C ratios ranged between 1 at % and 2 at %.

**Figure 3:**
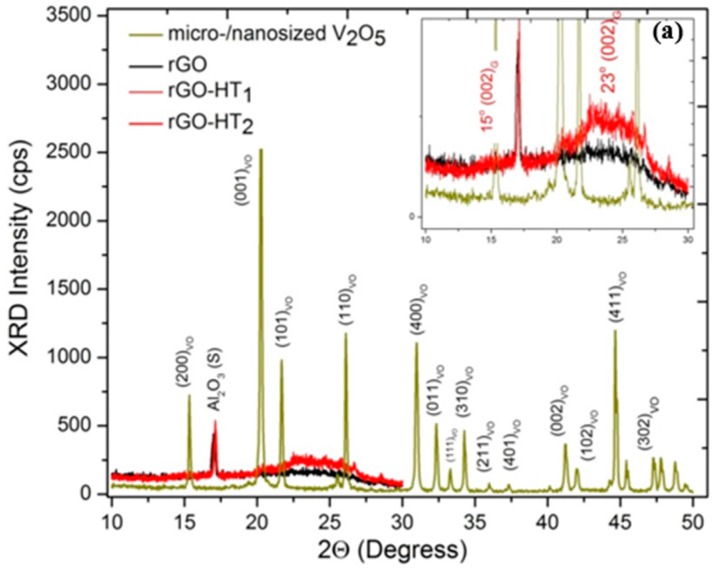

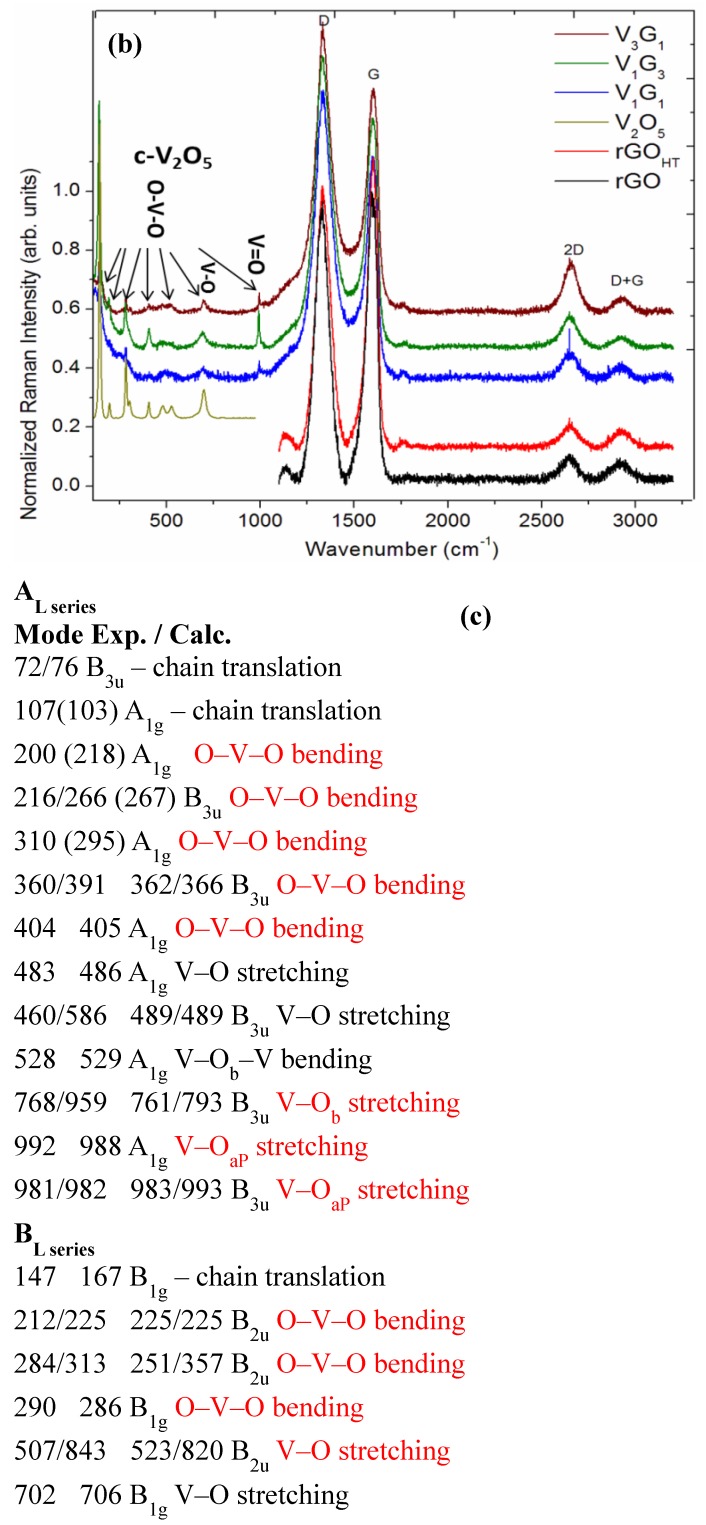
(**a**) X-ray diffractograms displaying characteristic peaks for graphene (002) and VNBs along with the substrate (Al_2_O_3_). The narrow peaks exhibit higher crystallinity and their position is indicative of primarily the α-V_2_O_5_ phase; (**b**) Micro-Raman spectra showing peaks associated with rGO and V_2_O_5_. The characteristic D, G, 2D and D + G combination bands are apparent along with V_2_O_5_ nanobelts for composites; (**c**) Experimental and calculated Raman spectral bands for the V_2_O_5_ lattice are also tabulated with group symmetry.

The crystalline structures of pristine rGO_HT_, VO and GVNBs are investigated by X-ray diffraction (XRD), shown in Figure 3a. The peak of pristine rGO_HT_ is somewhere in between the GO precursor and the traditional graphene, and that of VO matched the corresponding bulk pattern (JCPDS Card No. 89-0612). Qualitatively, the relatively sharper peaks are reflective of the medium range order of the nanocrystalline grain size distribution from Debye–Scherrer’s equation [48]. The XRD patterns of different composites (V_3_G_1_, V_1_G_1_ and V_1_G_3_) of GVNBs contain peaks of GO, rGO_HT_ and VNBs. The peaks at 15.2°, 22.1°, 25.9°, 28.5°, 32.0° and 41.7° correspond to the (200), (101), (110), (111) and (002) planes of VNBs, respectively [49]. The interlayer distance of GVNBs at the (200) reflection (d_101_) was determined to be 0.584 nm [50,51]. The peak at 24.2° is assigned to partially-reduced rGO_HT_ [52]. Generally, GO is reduced to rGO via high-temperature heat treatment (thermally-reduced GO) or by strong reducing agents [53,54]. When GO is reduced by external factors, the GO peak position shifts towards higher diffraction angles, and the new peak appears at 24.2°, corresponding to the (002) plane with an interlayer distance of 3.67Å as the characteristic of rGO_HT_. Interestingly, the GVNBs show sharp characteristic peaks associated with both VO and rGO. Toward further gaining insights into the chemical structure of hybrids, we used nondestructive Raman spectroscopy (RS) to probe chemical structural bonding. Figure 3b displays first- and second-order Raman spectra in the spectral region 150–3200 cm^−1^ for all of the samples studied. For a realistic comparison, the spectra are normalized to the intense spectral band at a wavenumber of ~1595 cm^−1^. Raman spectra show characteristic crystalline VO peaks (marked) appearing at 218, 267, 310, 360, 486, 529, 586, 768 and 982 cm^−1^ ascribed to the A_1g_, B_1g_ and B_3u_ normal modes of V_*x*_O_*y*_ (see Figure 3c, box) [55,56,57,58]. The difference in band position for different composites is apparent and may be attributed to the lattice crystal structure, level of oxygenated (G–V and V–O) bonding and graphene-metal oxide interactions. The two characteristic diagnostic peaks centered at 1370 cm^−1^ (disorder-induced D band) and 1595 cm^−1^ (in-plane stretching or tangential G band) correspond to the breathing mode of κ-point phonons of A_1g_ symmetry and the first-order scattering of E_2g_ phonons of graphene, respectively. The other Raman peaks of interest are second-order bands at 2670 cm^−1^ and 2920 cm^−1^ assigned to the 2D band and a combination of the D + G band, respectively [59,60]. All of the structural characterization results indicate that GNS and VO-related materials coexist in the prepared hybrids successfully. While the XRD revealed bulk phases, the Raman spectra allow identification of local and surface vanadium pentoxides species, which have a strong influence on the electrochemical reactivity and energy storage discussed below.

### 3.2. Electrochemical Properties and Impedance Data Simulation

**Figure 4:**
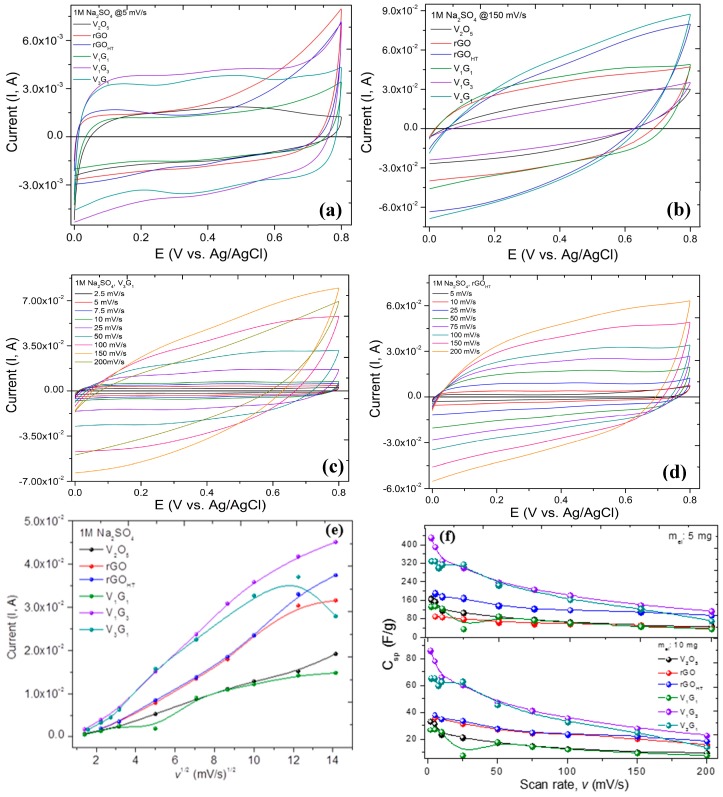
(**a**–**d**) Potentiodynamic (cyclic voltammetry; CV) mode showing quasi-rectangle behavior with redox peaks at a smaller scan rate; analyses in terms of (**e**) current versus *sqrt* scan rate and (**f**) gravimetric specific capacitance (*C*_sp_; F·g^−1^) with scan rate.

**Figure 5:**
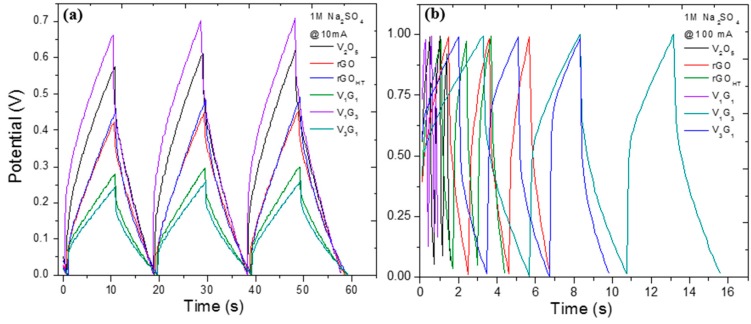
Galvanostatic charging-discharging profiles between 0.01 and 1.0 V at current densities of (**a**) 1 and (**b**) 10 A·g^−1^ showing characteristics discharging capacitor shapes.

Figure 4a–d shows cyclic voltammograms for all of the GVNB composites at scan rates of 5 and 150 mV/s and V_3_G_1_ and rGO_HT_ with scan rates ranging from 2.5 to 200 mV/s, in 1 M Na_2_SO_4_ electrolyte. The CV curves show a quasi-rectangular behavior, confirming the double-layer capacitance of these composite electrodes. The vanadium-rich composite (V_3_G_1_) shows broader redox peaks with retention of the quasi-rectangular shape in the potential window of 0.0–+0.8 V. The CV from graphene derivatives are in stark contrast to the hybrids, which are nearly rectangular loops, indicative of almost an ideal supercapacitor. Specifically, all hybrid electrodes displayed well-defined pseudocapacitive behavior at a scan rate of 5 mV/s with two redox peaks at +0.18/+0.3 V (cathodic I/anodic II) and +0.45/+0.5 V (cathodic III/anodic IV) related to presumably faradaic (redox) reactions relating to the conversion between different vanadium oxidation states. Moreover, the VNBs have a layered crystal structure and multivalent oxidation states of vanadium ions. These properties facilitate the insertion and extraction of alkali-metal electrolyte ions (Li^+^, Na^+^, K^+^, etc.) in the vicinity of the electroactive material. The electrochemical sodium-ion insertion process can be expressed as follows [61,62]: $V_{2}O_{5}+x{\text{Na}}^{+}+xe^{-}↔V_{2-x}^{5+}{\text{Na}}_{x}^{+}V_{x}^{4+}O_{5}^{2-}+x{\text{Na}}^{+}$. From the equation, the charging-discharging processes involve reversible intercalation of Na^+^ into layered VNBs with simultaneous electron transfer, i.e., the partial reduction of V^5+^ to V^4+^ (and vice versa during oxidation), and thus, provides pseudocapacitance to V_*x*_G_*y*_ composites. Figure 4e shows the variation of the maximum current with the square root of the scan rate (*v*^1/2^), and the quasi-linear behavior, especially at higher scan rates, is reminiscence of diffusion-limited (mass transport) phenomena and reflective of heterogeneous diffusion attributed to the composite nature of hybrid electrodes. The magnitude of the current observed is governed by the Randles–Ševćik equation for a reversible transfer process, $I_{rev}=0.446\;FAC{\left(FDv/RT\right)}^{0.5}$ or $I_{rev}=\left(2.69\times 10^{5}\right)n^{3/2}{ACD}^{1/2}v^{1/2}$, alternatively, representative of either a multielectron quasi-reversible (most likely for our hybrids) or a case of a fully-irreversible electron transfer process following: $I_{irrev}=0.469{\left(\alpha n^{\prime }\right)}^{0.5}nFAC{\left(FDv/\text{RT}\right)}^{0.5}$ (or $I_{irrev}=\left(2.99\times 10^{5}\right)n{\left(\alpha n^{\prime }\right)}^{0.5}{ACD}^{1/2}\;v^{1/2}$), where *A* is the geometric area of the electrode (cm^2^), α is the transfer coefficient (usually presumed to be close to 0.5), *F* is the Faraday constant (C·mol^−1^), *D* is the diffusion coefficient (cm^2^/s), *C* is the concentration (mol/cm^3^), *v* is the scan rate (V/s), *R* and *T* are the usual constants, *n* (=1) is the total number of electrons transferred in the electrochemical process and *n*′ (=0.6) is the number of electrons transferred before the rate determining step. The analysis of the current helped to determine the *D* coefficient that ranged between 2 × 10^−9^ and 8 × 10^−9^ m^2^·s^−1^ for all of the samples studied. The larger integrated area CV curves for hybrids implies that they have higher specific capacitance (*C*_s_), as discussed below. The specific gravimetric capacitance (*C*_s_) of the hybrid electrodes is calculated according to the following equation: $\frac{1}{mv\left(V_{f}-V_{i}\right)}\int_{V_{i}}^{V_{f}}I\left(V\right)dV$, where *m* is the mass of the active electrode material (g) measured using a micro-balance. Figure 4f presents variation in *C*_s_ with the scan rate for all of the electrodes that show a gradual decrease in *C*_s_ with the scan rate peaking at a scan rate of 5 mV·s^−1^, being 450, 350 and 180 F·g^−1^ for V_1_G_3_, V_3_G_1_ and rGO_HT_, respectively, as an upper bound, considering that the average mass of the electrode material is 5 mg. We also measured charging-discharging (*V–t*) profiles shown in Figure 5 and determined gravimetric capacitance following: $C_{s}=2\times \frac{I\Delta t}{\Delta Vm}$, where I is the applied discharge current, Δ*t* is the discharge time after IR drop, Δ*V* is the discharge potential window after IR drop and m is the mass of the single electrode material, respectively. The factor of 2 is used because series capacitance is formed in a two-electrode cell system. The *V*–*t* profiles between 0 and 1.0 V at different current densities and cyclability of various electrodes exhibits stable performance for more than 500 cycles, and the fading in *C*_s_ is possibly due to internal resistance and polarization of the electrodes. The *C*_s_ values are comparable at a specific current density 10 A·g^−1^ to those determined using CV curves. Moreover, *C*_s_ of the hybrid electrodes was significantly larger than that of rGO-only electrodes with values of ~60–70 F·g^−1^. Generally, the rate capability is strongly influenced by ion diffusion in the electrolyte, the surface adsorption of ions on the electrode materials (electrode/electrolyte accessibility) and the charge transfer in or on the electrode. At higher scan rates, any of these three processes is relatively slower, which limits the rate, lowering the *C*_s_. Nevertheless, the higher specific capacitance is attributed to smaller size vanadium pentoxides nanobelts, resulting in high specific capacitance. Although GNS have a supplementary (supercapacitive) contribution to the hybrid composites, they have excellent electronic conductivity and, thus, shuttle the electrons between vanadium-oxide nanostructures and the current collector. Therefore, the chemical integration between rGO_HT_-vanadium pentoxide nanobelts (VNB) into a single system enhanced the electrochemical behavior of pseudocapacitive electrodes. The synergistic effects of chemical bridging (utilizing electrostatic and coordination interactions between negatively-charged surface functional groups of (rGO) and V^4+^/V^5+^ ions), the crumpled and flower-like surface morphology promoted tailored properties and interfaces and topologically interconnected network architectures [63,64,65]. We attribute this enhancement to the concomitant double-layer or non-Faradaic capacitance and pseudocapacitive (redox) electrochemical processes on the addition of rGO with VNBs (V_m_G_n_) composite materials. Following the $V_{2}O_{5}+x{\text{Na}}^{+}+xe^{-}↔V_{2-x}^{5+}{\text{Na}}_{x}^{+}V_{x}^{4+}O_{5}^{2-}+x{\text{Na}}^{+}$ reaction equation, the charging-discharging processes involve reversible intercalation of Na^+^ into layered VNBs with simultaneous electron transfer, i.e., the partial reduction of V^5+^ to V^4+^ (and vice versa during oxidation) and, thus, provide pseudocapacitance to V_m_G_n_ composites. It is important to note that the composite electrodes possess higher *C*_s_ values than those of the constituents.

**Figure 6:**
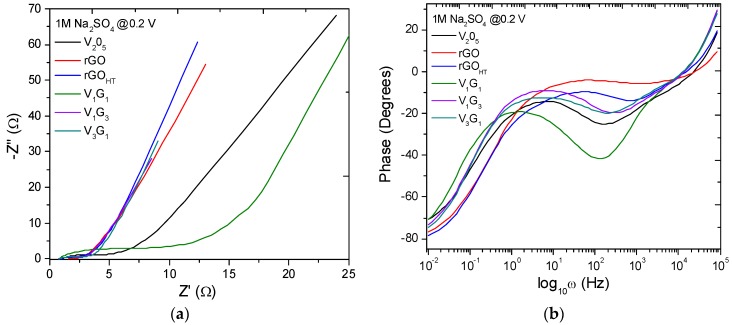

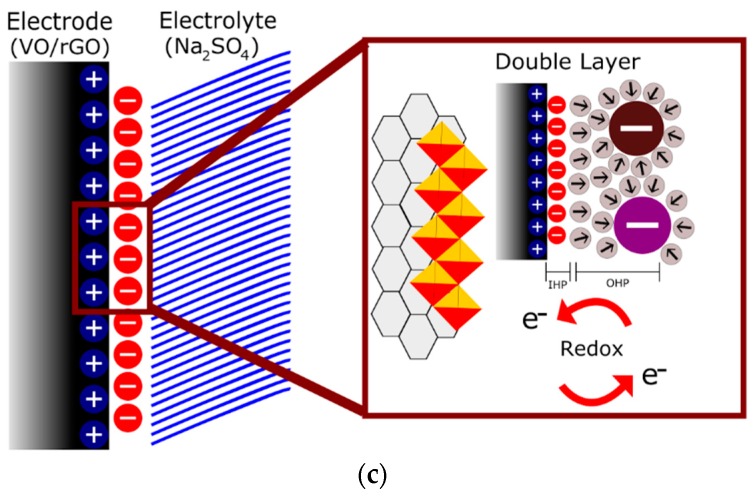
(**a**) Nyquist plots; (**b**) phase behavior with equivalent circuits; and (**c**) electrode/electrolyte interfacial schematic.

**Figure 7:**
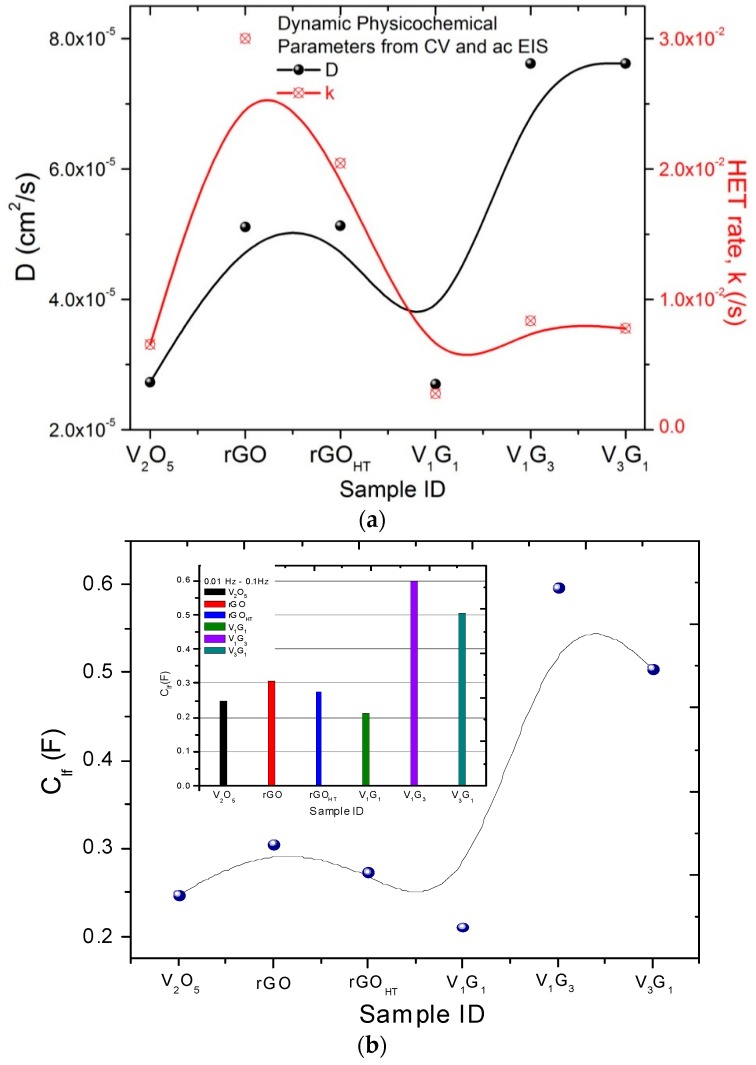
Variation of (**a**) *D* and *k* and (**b**) low frequency capacitance *C*_lf_. The inset shows *C*_lf_ as a histogram.

**Figure 8:**
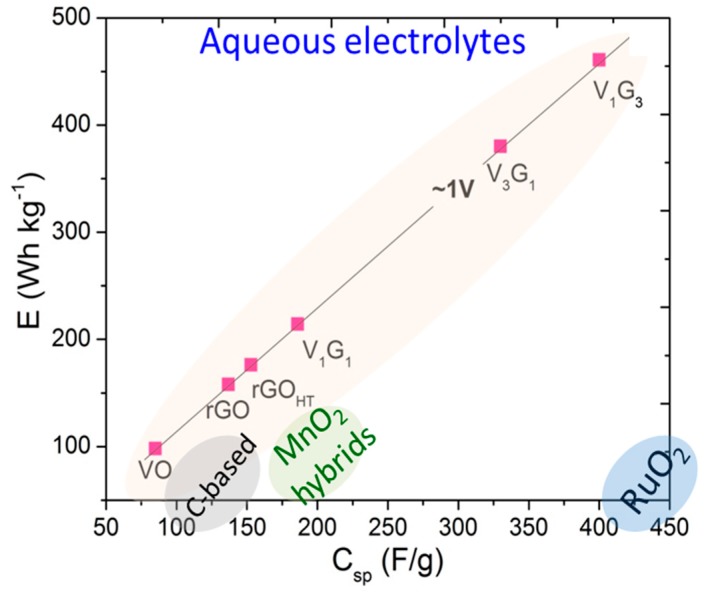
Specific energy density versus specific capacitance behavior along with other relevant materials in aqueous electrolyte for comparison.

In order to further understand the reasons behind the unique performance of hybrid electrodes, electrochemical impedance spectroscopy (EIS) data were analyzed to investigate electrode kinetics and to quantify electronic and ionic contributions besides determining various circuit elements in bulk electrolyte and at the electrode/electrolyte interface. Nyquist plots (−*Z*″ versus *Z*′) are shown in Figure 6a for representative hybrids besides the phase response with semilog frequency in Figure 6b. The impedance plots exhibit good supercapacitor behavior with a straight sloping line (i.e., solid-state diffusion in bulk electrolyte and OH– ion diffusion or electron transport into the electrode) in the low-frequency (interfacial) region and a small semicircle arc (i.e., solid-electrolyte interphase and grain boundary) in the high-frequency region (see Figure 6c for the electrode/electrolyte interfacial schematic). Furthermore, the phase change of almost 70°–80° indicates the capacitive behavior of the hybrids. The impedance data were simulated to fit the experimental data using in-built “sim” software with our electrochemical workstation with different equivalent circuit models and to extract useful circuit parameters. Figure 6a,b insets show proposed equivalent circuit models, and the simulated results are summarized in Table 2 and Table 3, respectively. The models consist of (1) *R*_s_, equivalent series resistance (ESR), which includes ionic or bulk resistance of the electrolyte solution combined with interface resistance, intrinsic and contact resistance at the electroactive material/current collector [66], *R*_ct_, charge transfer resistance, *Z*_W_, Warburg impedance, *C*_dl_, double layer capacitance, as in Randles’ equivalent circuit; and (2) the constant phase element (CPE) equivalent circuit [67,68,69]. The CPE circuit includes an inductor (*L*_s_) to account for self-inductance of the wire leads, a capacitive element (*C*_e_), solution (*R*_s_) and charge transfer resistance (*R*_ct_), Warburg impedance (*Z*_W_) and CPE (*Q*_0_, *n*), replacing the capacitor used to characterize double-layer capacitance (*C*_dl_) in Randles’ circuit. By definition [67], 1/*Z*_CPE_ = *Y*_o_ = *Q*_o_ (ω)*^n^* e^-iπ*n*/2^; *Q*_o_ = 1/|*Z*| at ω = 1 rad/s, *n* = 1 is for an ideal capacitor and *n* = 0, a pure resistor. In principle, the ESR from Circuit Model 1 determined from the high-frequency intercept at real *Z*′ is smaller for the composite hybrid electrodes (<1.5 Ω) than those of only component electrodes (see Table 2 for a summary of circuit parameters) [70]. The broad arc in the high-frequency region corresponds to the charge transfer resistance (*R*_ct_) caused by the pseudocapacitive behavior of electroactive material (electrode), double-layer charging on the electrode surface (*C*_dl_) and Warburg impedance (*Z*_W_) [71]. The inductance in the CPE circuit is estimated to be ~700 nH [72], and therefore, for example, at 98 kHz, the electrode leads contribute 0.43 Ω toward the complex impedance. Studying the CPE behavior, Hirschorn et al. [67] assume time-constant dispersion as a result of the distribution of capacitance and/or charge-transfer resistance across the electrode surface, presumably originated from non-uniform electrode thickness and porous surface geometry. Furthermore, the inclusion of a single RC element in the circuit model reduced its accuracy, leading to the inclusion of a series capacitor to describe the time-constant distribution. The capacitive element (*C*_e_) is representative of the dispersion of time-constants in the samples (either through *C* and/or *R* in series or in parallel). The dimensionless parameter *n* in Table 3 for all of the materials is nearly 0.65, suggesting that there is a resistivity distribution in compliance with our presumption of the non-uniform electrode thickness, loading or weight distribution of VNBs on rGO_HT_ and the porous network. Figure S1 provides representative simulated fits to experimental impedance spectroscopy data for rGO_HT_, V_2_O_5_, V_1_G_3_ and V_1_G_1_. For the macroelectrode and diffusion layer of infinite thickness, Z_W_ = R_ct_ λ/(ω)^1/2^ and $\lambda =\frac{k_{ET}^{f}}{\sqrt{D_{f}}}+\frac{k_{ET}^{r}}{\sqrt{D_{r}}}\approx \frac{k_{ET}}{2\sqrt{D}}$, where *k*_ET_ is the heterogeneous kinetic rate constant on the electrode and *D* is the diffusion coefficient of the redox species. The increasing slope trend exhibits the capacitive nature related to the film charging mechanism that is typically characteristic for mesoporous electrodes. The relatively lower *R*_ct_ values for all of the hybrid electrodes studied reflect an enhancement in the electronic and ionic conductivities of vanadium pentoxides adsorbed on or intercalated within GNS (see Figure 7a for the variation of *D* and *k*). Finally, the slope of the Nyquist plots in the low frequency region tends to increase with the presence of underlying graphene support films, reflecting a decrease in the Warburg resistance (*Z*_W_) or faster electrolyte ion diffusion into the hybrids. Figure 7b and the inset histogram show low frequency capacitance *C*_lf_ variation derived from the following relationship: $\frac{1}{C_{\text{lf}}\left(\omega \right)}=\frac{Z^{\prime \prime }\left(\omega \right)}{1/\omega }$. Once again, the *C*_lf_ peaks for V_1_G_3_ and V_3_G_1_ hybrid electrodes are consistent with the results obtained above using CV and galvanostatic measurements. The presence of rGO_HT_ creates more electroactive sites and tailored interfaces, in turn facilitating the access of electrolyte ions and making electron transport between GNS and VNBs easier. This corroborates the presumption that these hybrids can be electrically simulated to an assembly of parallel RC omponents normal to the electrode surface. The energy density $E=\frac{1}{2}\frac{C_{s}V^{2}}{m}\frac{1000}{3600}$ and power density $\left(P=\frac{E}{\Delta t}=\frac{I\Delta V}{2m}=\frac{V^{2}}{4mR_{s}}\;\times 1000\right)$ for each of these hybrid electrodes are plotted following specific energy versus specific capacitance and compared to other hybrid nanomaterials (Figure 8). These results suggest that the presence of GNS as a support for hybrid electrodes not only improved the electrical conductivity and mechanical stability, but also served as a functional charge transfer-like dopant to or from the vanadium-based nanomaterial discussed below.

### 3.3. Density Functional Theory Simulations

**Figure 9:**
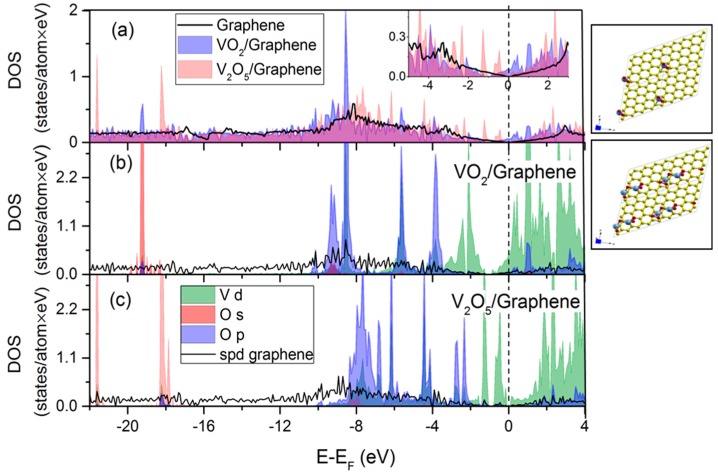
Density functional theory (DFT) calculations. Total electronic density of state (DOS) spectra per atom eV for (**a**) clean graphene, VO_2_/graphene, V_2_O_5_/graphene (4 × 4 graphene supercell); partial DOS spectra of (**b**) VO_2_/graphene and (**c**) V_2_O_5_/ graphene for V d, O 2s and O 2p orbitals and spd graphene. Schematics of graphene, vanadium oxide and vanadium pentoxide clusters on the 4 × 4 graphene supercell (top view, right panel). The vertical line is the Fermi level.

Density functional theory (DFT) ab initio calculations help to elucidate the fundamental properties at graphene-metal oxide and graphene oxide-metal oxide interfaces, particularly in terms of lattice stability, electronic structure (electron density of states (DOS)) [73] and interfacial/surface charge transfer. Schematic diagrams for the clean graphene support, clusters of adatoms VO_2_ and V_2_O_5_ on the graphene sheet (top view, right panels of Figure 9) alongside comparative total and partial DOS spectra of the VO_2_/graphene and V_2_O_5_/graphene (the most stable structure) are shown in Figure 9a–c, quantifying unoccupied and occupied states. From the electronic DOS behavior, the hybrids exhibit semi-metallic behavior, i.e., finite DOS energy levels within the Fermi level, making a considerable contribution to the total DOS at *E*_F_, and this behavior changes marginally with different graphene-vanadium oxide (VO_2_ and V_2_O_5_) interfaces, contributing toward enhanced electroactive sites. The partial DOS calculations also show that the electron contributions of V d states prevail and the other contributions of the 2s and 2p oxygen states. Table 4 provides the summary of the adsorbate orbitals per atom and the surface charge transfer (alternatively, doping type) between the adsorbate and the graphene support [40]. More specifically, adsorption of VO_2_ on graphene causes charge transfers from the adsorbates to the substrate of 0.1 *e* and 0.19 *e*, respectively. The exact opposite is observed for V_2_O_5_ on graphene. For VO_2_ and V_2_O_5_ on graphene, some charge from the vanadium 4s orbital is transferred to the empty 4p orbital. Additionally, for V_2_O_5_ (the vanadium average charge is 21.6 *e*), we have four oxygens with a charge of about 8.5 *e*, whereas the fifth oxygen has a charge of about 9 *e*. On the contrary, for VO_2_ (vanadium average charge is 21.8 *e*), the charge for oxygens is about 8.5 *e*. This implies that the fifth oxygen in V_2_O_5_ is pulling electrons from the substrate. This is consistent with the statement that the graphene conductivity is never smaller than the minimum value of the quantum conductivity limit. Thus, it is possible to suppose that graphene can retain its unique properties in graphene/V_*x*_O_*y*_ systems similar to other transition metal oxide-graphene interfaces [4,13,14]. Moreover, it is noteworthy that higher performance originates not only from the Gr/VO interactions, but also arises from the dimensional tuning of V_*x*_O_*y*_ at the nanoscale. It is conceivable that nanostructures and the thin layer being accessible to the electrolyte result in the maximum utilization of the metal oxides (i.e., minimization of dead volume).

## 4. Conclusions

In summary, a series of graphene nanosheets/vanadium pentoxide nanobelt hybrids with different weight ratios was prepared by a facile hydrothermal decomposition method. We systematically performed microscopic structural and electrochemical properties/performance studies. The dimensionality of graphene nanosheets combined with vanadium pentoxides creating tailored interfaces with tunable properties plays a significant role and is a key factor in determining the response of these materials towards electrochemical (re)activity. The electrochemical properties showed higher specific capacitance for graphene-rich composites with optimized VO loading as compared to VO-rich ones and the constituents, and this decreased with increasing scan rate, as anticipated. We attribute this to the fact that the nanostructured VO is involved in the faradaic reaction (pseudocapacitance) coupled with highly conducting rGO_HT_ having a large surface area, implying supercapacitive behavior, which can be practically used to deliver high power and energy density along the Ragone plot metrics. The three-dimensional multiplexed and highly conductive pathways provided by the rGO_HT_ scaffold architectural support also ensure rapid charge transfer and conduction due to the larger accessible geometric surface area. We simulated *ac* impedance spectroscopy data with conventional and CPE circuit models, which was useful in determining various equivalent circuit parameters. We attribute the overall good performance due to the synergistic effects from supercapacitive graphene as an elastic and electrically-conductive matrix and pseudocapacitive VNBs, allowing faster ion transport across the electrolyte.

## Figures and Tables

**Table 1:** Deposition parameters for the hydrothermal process. rGO_HT_, hydrothermally-processed reduced graphene oxide.

Electrode Material	V_2_O_5_ Mass (mg)	GO Mass (mg)	Total Mass (mg)	Temperature (°C)/Time (h)	Concentration (mg/mL)
rGO_HT_	0	20	20	180/6	0.5
V_1_G_1_	20	20	40	120/24	1
V_1_G_3_	10	30	40	120/24	1
V_3_G_1_	30	10	40	120/24	1

**Table 2:** Summary of ac impedance spectroscopy data simulations with Randel’s circuit element parameters.

Electrode Material	*R*_s_ (Ω)	*C*_dl_ (F)	*R*_ct_ (Ω)	*Z*_W_ (Ω)	*D* (cm^2^·s^−1^)	*k* (s^−1^)	Error (%)
VO	0.593	0.163	2.60	0.017	2.73 × 10^−5^	6.55 × 10^−4^	8
rGO	0.780	0.204	1.50	0.023	5.11 × 10^−5^	30.0 × 10^−3^	4
rGO_HT_	0.002	0.178	1.52	0.023	5.13 × 10^−5^	20.45 × 10^−3^	7
V_1_G_1_	0.270	0.082	0.110	0.120	2.70 × 10^−5^	27.73 × 10^−3^	11
V_1_G_3_	0.091	0.311	0.870	0.107	7.62 × 10^−5^	8.36 × 10^−3^	11
V_3_G_1_	0.145	0.270	0.780	0.054	7.62 × 10^−5^	7.77 × 10^−3^	10

**Table 3:** Summary of ac impedance spectroscopy data simulations with CPE circuit element parameters.

Electrode Material	*R*_s_ (Ω)	*C*_e_ (F)	*R*_ct_ (Ω)	*Q*_o_ (Ω/s)	*n*	*Y*_o_ (Ω^−1^)	*Z*_W_ (Ω)	Error (%)
VO	0.955	0. 304	4.170	0.0060	0.623	0.170	4.17	0.017
rGO	1.519	0.345	0.678	0.0035	0.650	0.311	2.27	0.023
rGO_HT_	0.937	0.323	1.199	0.006	0.653	0.259	2.73	0.023
V_1_G_1_	0.702	0.257	10.40	0.0057	0.665	0.160	4.43	0.120
V_1_G_3_	0.766	0.692	1.756	0.0057	0.687	0.527	1.34	0.107
V_3_G_1_	0.777	0.581	2.156	0.1145	0.626	0.462	1.53	0.054

**Table 4:** Charge changes (or transfer) between adsorbates (adatoms) and graphene monolayer support relative to free atoms and clean graphene.

Structure	Charge Changes		
	Adsorbate Orbital Populations Per Atom		Overall Charge Transferred to Graphene Support
**O/Graphene ^1^**	-	O-2s = 1.92 *e*	−0.56 *e*
	-	O-2p = 4.64 *e*	-
**VO_2_/Graphene ^2^**	V-4s = 0.05 *e*	-	0.19 *e*
	V-4p = 0.29 *e*	O-2s = 1.96 *e*	-
	V-3d = 3.48 *e*	O-2p = 4.47 *e*	-
**V_2_O_5_/Graphene ^2^**	V-4s = 0.06 *e*	-	−0.07 *e*
	V-4p = 0.21 *e*	O-2s = 1.95 *e*	-
	V-3d = 3.33 *e*	O-2p = 4.57 *e*	-

Note: calculations on ^1^ 2 × 2 and ^2^ 4 × 4 graphene supports.

## Supplementary Materials

The following are available online at www.mdpi.com/1996-1944/9/8/615/s1.
