# Supplementary material for: Vanadium Pentoxide Nanobelt-Reduced Graphene Oxide Nanosheet Composites as High-Performance Pseudocapacitive Electrodes: ac Impedance Spectroscopy Data Modeling and Theoretical Calculations
Source: Materials (Basel). 2016 Jul 25;9(8):615. doi: 10.3390/ma9080615 (PMC5509009; doi:10.3390/ma9080615)
Supplement: Supplementary file 1 [file materials-09-00615-s001.pdf]

Supplementary Materials: Vanadium Pentoxide Nanobelt-Reduced Graphene Oxide Nanosheet Composites as High-Performance Pseudocapacitive Electrodes: *ac* Impedance Spectroscopy Data Modeling and Theoretical Calculations

Sanju Gupta, Bryce Aberg, Sara B. Carrizosa and Nicholas Dimakis

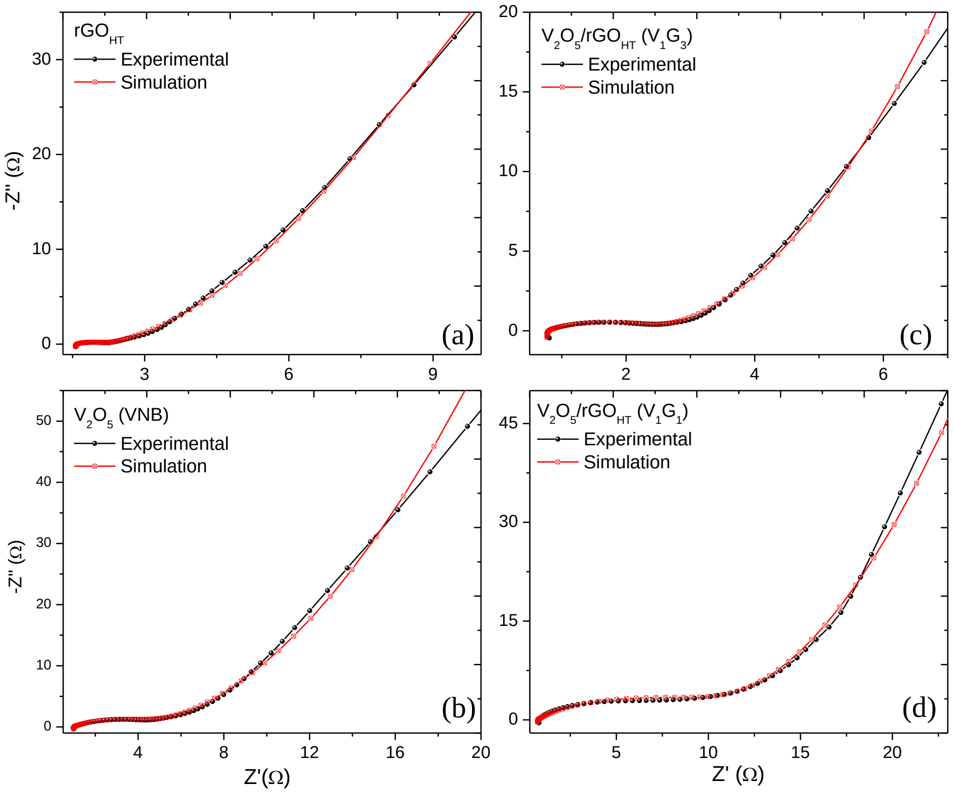

Figure S1. (Color online). Representative simulated fits to experimental impedance spectroscopy data for (a) rGO_{HT} (b) V_2O_5 (c) V_1G_3 and (d) V_1G_1.
